# Laser Light Trapping Phenomenon in a 3D Radiotherapy Polymer Gel Dosimeter

**DOI:** 10.3390/ma14143961

**Published:** 2021-07-15

**Authors:** Marek Kozicki, Malwina Jaszczak, Mariusz Dudek, Piotr Maras

**Affiliations:** 1 Department of Mechanical Engineering, Informatics and Chemistry of Polymer Materials, Lodz University of Technology, 90-543 Lodz, Poland; malwina.jaszczak@p.lodz.pl; 2 Institute of Materials Science and Engineering, Lodz University of Technology, 90-924 Lodz, Poland; mariusz.dudek@p.lodz.pl; 3 Medical Physics Department, Copernicus Hospital, 93-513 Lodz, Poland; piotr.maras@wp.pl

**Keywords:** laser light trapping phenomenon, VIC-T, 3D radiotherapy dosimetry, polymer gel dosimetry, laser light induced polymerization

## Abstract

This paper aims to explain the phenomenon of laser light trapping (LLT) in a 3D polymer gel dosimeter. A VIC-T polymer gel dosimeter containing 17% *N*-vinylpyrrolidone, 8% *N*,*N*′-methylenebisacrylamide, 12% tert-butyl alcohol, 5% gelatine, 0.02% hydroquinone and 14 mM tetrakis(hydroxymethyl)phosphonium chloride was used in this study. It was exposed to green laser light with a wavelength of 532 nm. A film was recorded during the exposure. After exposure, Raman spectroscopy was used to study the reactions taking place inside the dosimeter. The obtained results were used to explain what the LLT phenomenon is, what are the consequences for the dosimeter in which such a phenomenon occurs, and what dosimeter components play an important role in the occurrence of LLT. In addition, the conditions under which 3D polymer gel dosimeters can be measured using optical computed tomography at short wavelengths of visible laser light are indicated.

## 1. Introduction

Early approaches to measuring the dose distribution of ionizing radiation date back to the 1950s [1,2]. However, only after the development of magnetic resonance imaging (MRI) scanners and computers was it possible to switch to advanced 3D dosimetry, as proposed by Gore et al. [3], using a Fricke dosimeter [4] inserted into a polymer matrix with MRI reading. Subsequently, a number of other proposals for various 3D dosimetry systems were examined with the following readings: MRI, computed tomography (CT), ultrasonography (US) and optical computed tomography [5,6].

Among the various achievements in the field of 3D radiotherapy dosimetry, the one concerning the use of deoxidizers instead of infusion of dosimetric compositions with inert gas can be mentioned [7,8,9,10,11]. The principle of using oxygen scavengers is the binding of free oxygen in the 3D dosimetric solution by ascorbic acid into organometallic complexes in the presence of copper cations [7,8,12]. In addition other scavengers/antioxidants can be used for the same purposes for the preparation of normoxic dosimeters, such as tetrakis(hydroxymethyl)phosphonium chloride (THPC) [8,13,14,15].

This work concerns the phenomenon of laser light trapping occurring in the VIC-T 3D radiation therapy dosimeter, which contains the aforementioned THPC scavenger [16]. The dosimeter belongs to the family of 3D radiation therapy dosimeters containing *N*-vinylpyrrolidone (NVP). A summary of all NVP dosimeters and their recent applications is described elsewhere [16,17,18,19,20]. The results of the VIC-T [16] provided an optimal new composition of the gel dosimeter containing NVP, demonstrated the broad dose-response characteristics and thermal stability of the VIC-T, and indicated chemical (THPC-induced) and physical cross-linking of gelatine occurring in parallel after the manufacture of the dosimeter. During the study of the chemical and physical cross-linking of gelatine in the VIC-T, the phenomenon of laser light trapping was observed (see Figure 10 in [16]). The phenomenon manifested in the extinction of the scattered light recorded at right angle to the incident laser beam passing through the VIC-T dosimeter after a few minutes after VIC-T preparation and exposure to light. The scattered light intensity dropped to almost zero for some time, followed by an increase and a further decrease in the intensity. However, this phenomenon has not been fully recognized and investigated in [16].

Optical computed tomography has been shown to be very promising in the measurement of both 3D polymer gel and radiochromic gel dosimeters [21,22,23,24]. These early studies by Oldham et al. [21,22] on 3D polymer gel dosimetry resulted in sensitizing other researchers to optical artifacts and geometric distortions using optical computed tomography as a reading method. In this work, we indicate another potentially negative factor that may affect the use of short-wavelength laser beams for 3D scanning of some polymer gel dosimeters. This is due to the susceptibility of 3D gel dosimeters to daylight (broad UV-Vis spectrum), which has been observed with at least some such systems during typical routine laboratory production. However, no systematic and specific experiments have been carried out to find out which ingredients in each gel dosimeter formulation are responsible for their higher or lower sensitivity to daylight. Additionally, no such studies have been conducted to obtain knowledge of what wavelengths of light are conducive to the observed instability of gel dosimeters and what is the wavelength limiting at which dosimeters are insensitive to light. This work answers some of these questions.

The present work aims to provide an analysis of the laser light (532 nm) trapping phenomenon (LLT) and to elucidate the effect observed and reported elsewhere [16] for the VIC-T gel dosimeter. Conclusions were drawn related to the consequences of the LLT phenomenon on the use of similar dosimeters with optical computed tomography readings.

## 2. Materials and Methods

### 2.1. Preparation of VIC-T

VIC-T was prepared according to the preparation procedure developed in the previous study [16]. VIC-T is an acronym that originates from VIPAR^CT^, where “CT” stands for computed tomography, and VIPAR^CT^ has been abbreviated to VIC [11], whereas “-T” in VIC-T corresponds to tetrakis(hydroxymethyl)phosphonium chloride used in this composition as oxygen scavenger. VIC-T consisted of 17% (*w/v*) *N*-vinylpyrrolidone (NVP, Sigma Aldrich, St. Louis, MO, USA), 8% (*w/v*) *N*,*N*′-methylenebisacrylamide (MBA or Bis, Sigma Aldrich, St. Louis, MO, USA), 5% (*w/v*) gelatine (type A, 300 Bloom, Sigma Aldrich, St. Louis, MO, USA), 12% (*w/v*) tert-butyl alcohol (t-BuOH, Sigma Aldrich, St. Louis, MO, USA), 0.02% (*w/v*) hydroquinone (HQ, Riedel-de Haen, Seelze, Germany) and 14 mM tetrakis(hydroxymethyl)phosphonium chloride (THPC, Sigma Aldrich, St. Louis, MO, USA). Initially, HQ was added to distilled water at room temperature and mixed thoroughly. Then MBA was added and heated to 45 °C. Gelatine was then added and allowed to dissolve. After 15 min of stirring, the solution was equilibrated at 32 °C and NVP was added. t-BuOH was added at 35 °C and the temperature was raised to 40 °C to dissolve all ingredients. The solution was then cooled to 30 °C and THPC was added with stirring for 30 s. The prepared dosimetric solution was poured into a quartz round bottom vial (dimensions: diameter 2.5 cm, height 7.5 cm) for static light scattering measurements (Section 2.2). The solution was also used to fill a quartz cuvette (dimensions: 4.2 × 4.5 × 0.9 mm^3^; see Figure 6) for Raman spectroscopy measurements (Section 2.3). VIC-T without HQ and VIC-T without THPC in the composition were also prepared in quartz round bottom vials for static light scattering measurements.

### 2.2. Static Light Scattering (SLS) Measurements

**Figure 1:**
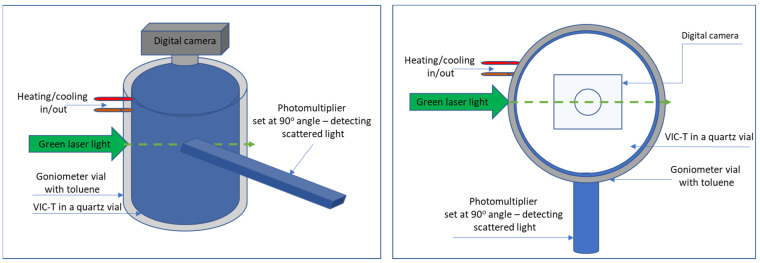
A scheme illustrating the experiment set up; left—side view, right—top view of the SLS-Systemtechnik goniometer with VIC-T dosimeter vial inside the goniometer’s vessel with toluene; a photomultiplier set at 90° angle with respect to a green (532 nm, 5 mW) laser light passing through VIC-T; a digital camera set at the top of the instrument’s goniometer above a niche inside which VIC-T is placed.

The VIC-T was irradiated with green laser light of a static light scattering instrument (SLS-Systemtechnik, Freiburg, Germany, equipped with a Uranus DPSS 532 nm, 5 mW laser). The instrument’s laser power was kept constant throughout the measurement. At the same time, the instrument measured the intensity of the scattered light at an angle of 90° (angular measurements are possible in the range of 15–145° thanks to the classic design of the goniometer and the Hamamatsu R928 photomultiplier). Measurements were carried out at 23 °C for a maximum of 1 h after sample preparation. A film was recorded during the measurements. For this purpose, a camera (10-megapixel matrix, Canon A 20000 IS, Canon, Tokyo, Japan) was mounted on the goniometer reservoir containing the sample. A scheme illustrating the experiment set up is shown in Figure 1. Note that SLS measurements were also performed for VIC-T without HQ and VIC-T without THPC in the composition. In the case of VIC-T without THPC in its composition, the measurement temperature of the SLS had to be lowered to 19 °C to trigger the conversion of the VIC-T solution into a physical gel; VIC-T has a reduced concentration of gelatine (5%) compared to the previous VIC composition (7.5%) [11] due to the presence of THPC (see [16] for a detailed explanation), which makes it difficult for VIC-T without THPC to convert to physical gel.

### 2.3. Raman Spectroscopy

Raman spectra were acquired for a VIC-T quartz cuvette (Slinap, Lodz, Poland) (dimensions: 4.2 × 4.5 × 0.9 mm^3^; see Figure 6) after irradiation with green laser light (2 mW, 532 nm, He-Ne), as described in Section 2.2. For this purpose, a Raman spectrometer (inVia Renishaw, Gloucestershire, United Kingdom) was used, and the setting parameters were as follows: (i) laser-emitting light at 532 nm, (ii) laser power of 2.1 mW, (iii) spectral range of 100‒3200 cm^−1^, and (iv) exposure time in the range of 40 s. It should be noted that such a short exposure time should not convert the VIC-T monomers into a polymer (no such observations have been made).

### 2.4. Large Volume VIP in Stiff and Not Stiff Containers

In the experiment, a VIP polymer gel dosimeter was used, which was prepared as described elsewhere [9]. VIP abbreviation originates from VIPAR^nd^ [9], where “nd” stands for “normoxic, double” polymer gel dosimeter and VIP is a shorter acronym of the same dosimeter. It was prepared by dissolving 4% (*w/v*) MBA (Sigma Aldrich, St. Louis, MO, USA) in deionized water under stirring and heating below 50 °C. Next, 7.5% (*w/v*) gelatine (type A, 300 Bloom, Sigma Aldrich, St. Louis, MO, USA) was added and completely dissolved. The solution was then cooled down to a temperature of about 33 °C in order to add 8% (*w/v*) NVP (Sigma Aldrich, St. Louis, MO, USA). NVP was purified beforehand by distillation under reduced pressure (80 °C, 8 mbar). Finally, 0.007% (*w/v*) L-ascorbic acid (ASC, Chempur, Piekary Slaskie, Poland) and 0.0008% (*w/v*) copper sulfate pentahydrate (CuSO_4_ × 5H_2_O, Chempur, Piekary Slaskie, Poland) were added and the composition was mixed. After preparation, two cylindrical containers (PH-5-DD1, GeVero Co., Lodz, Poland) were filled. The outer diameter of the containers was 100 mm, the wall thickness was 5 mm and the height was 210 mm. One container is equipped with a pressure equalizing valve to eliminate stresses in the gel substance of the dosimeter; however, another container was produced without such a valve. The height of the irradiated part filled with gel was 130 mm. The vial was immersed in a water phantom (GeVero Co., Lodz, Poland) such that the center axis of the phantom coincided with the isocenter of the linear accelerator (Source to Surface Distance, SSD, 92.5 cm). Next, the dosimeter was irradiated with a photon beam (6 MV, 600 MU/min, Clinac 2300, X6, Varian, Palo Alto, CA, USA) to get three areas of two opposing open fields (4 × 4 cm^2^, 0 and 180°); a higher dose of 40 Gy (2500 MU per field), a lower dose of 20 Gy (1200 MU per field) and a field wedge of 60° (3700 MU) between these two doses to stimulate the dose gradient. After irradiation, the dosimeter in the two vials was visually assessed.

## 3. Results and Discussion

### 3.1. Effects inside VIC-T

The VIC-T 3D polymer gel dosimeter was previously tested [16] with a static light scattering apparatus (SLS) at two temperatures: 23 and 43 °C, at the right angle of the goniometer. The configuration of the instrument allowed for the registration of the scattered light quanta from the VIC-T during the experiment. Similar results were obtained at both temperatures (see Figure 10a,c in [16]). The main surprising observation was that after about 4 min from the start of the experiment, the intensity of the scattered light increased sharply, followed by a sharp drop to very low values, below the values recorded at the beginning of the experiment. The instrument recorded these low values of the scattered light intensity, regardless of the measurement temperature, for a period of 15 and 5 min, respectively, at a lower and higher temperature. The laser light seemed to be trapped, and the SLS detector at right angle to the incident laser beam did not register much light scattering. This observation was called the laser light trapping (LLT) phenomenon in the 3D polymer gel dosimeter.

**Figure 2:**
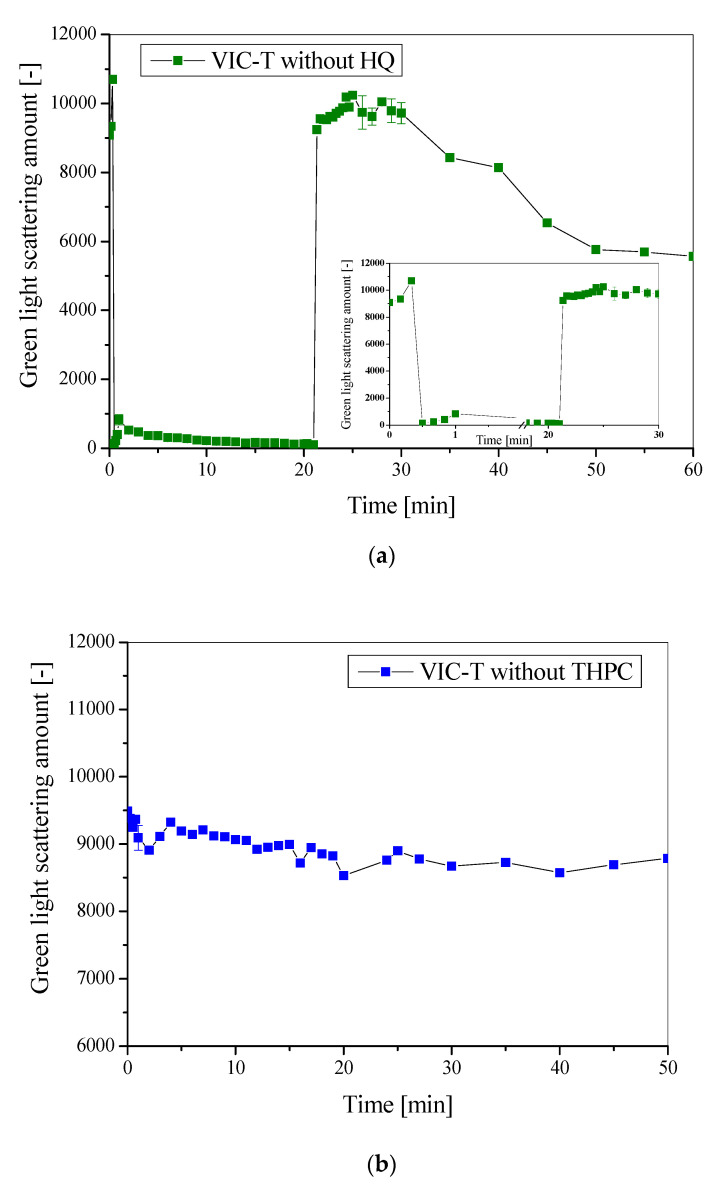
The green laser light (532 nm, 5 mW) scattering intensity (SLS) measurements at 90° for VIC-T over time after preparation: (**a**) is for VIC-T without HQ (at 23 °C) and (**b**) is for VIC-T without THPC (at 19 °C). Inset in (**a**) is for a narrower time range to visualize the initial period of measurements.

In the present work we undertook to repeat such measurements at 23 °C for the VIC-T. The results were as in the previous study [16]. In addition, we tried to answer the following questions: (i) which VIC-T components are necessary for the occurrence of the LLT phenomenon and (ii) what is actually behind it. Thus, SLS measurements were carried out for VIC-T without HQ and VIC-T without THPC in the composition, because in the previous work the VIC-T was examined without gelatine and without gelatine and THPC (see Figure 10a,c in [16]). For the latter two, the LLT was not noticed. Figure 2 shows the actual results for VIC-T without HQ and VIC-T without THPC. Figure 2a shows that HQ does not play a role in the LLT phenomenon as the phenomenon does not occur regardless of its presence or absence in the dosimeter composition. However, Figure 2b shows that THPC is important for the occurrence of LLT; without this, no LLT was observed. Therefore, by combining the experimental results of the previous work with the results of the present work, it is concluded that both gelatine and THPC should be present in a dosimeter composition for the LLT initiation. It also means that without THPC the VIC-T is stable under the influence of green laser light. Consequently, THPC itself or THPC covalently linked to gelatine [16] is susceptible to laser irradiation. At this point, it was realized that the exact mechanism behind this photochemically induced process required separate studies.

**Figure 3:**
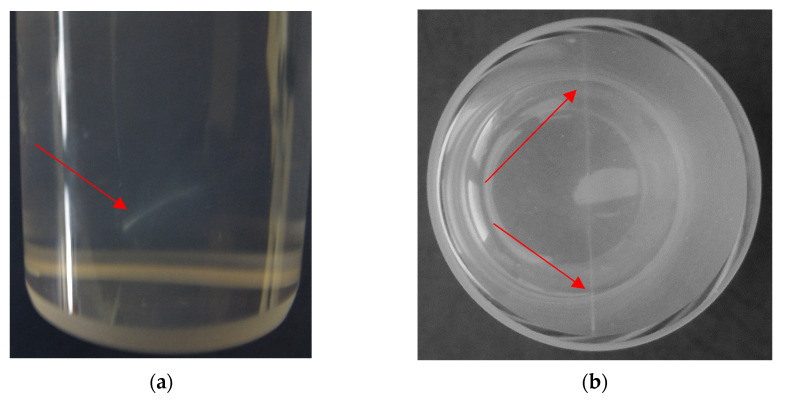
Side (**a**) and top (**b**) images illustrating the line—the effect of passing a green laser light of 532 nm (5 mW) through VIC-T polymer gel dosimeter. The white line across the shorter axis of the VIC-T vials (indicated with red arrows) corresponds to polymerization and crosslinking of monomeric components of VIC-T.

After the SLS measurements described above, the question posed above remained: what is behind the observed LLT phenomenon. It occurred that one important effect in VIC-T following exposure to green laser light was overlooked in former study [16]. Without realizing it, the VIC-T was disposed. On the other hand, in the present work, the dosimeter after laser irradiation was visually analyzed, which ended with noticing a very thin, barely visible white line passing through the gel substance exactly along the path of the green laser light (Figure 3). It has become apparent that exposure to this green laser light of certain 3D polymer gel dosimeters containing gelatine and THPC (VIC-T in our case) cause polymerization and cross-linking of the vinyl monomers in the dosimeter composition. However, this observation did not fully answer the question about the LLT phenomenon.

**Figure 4:**
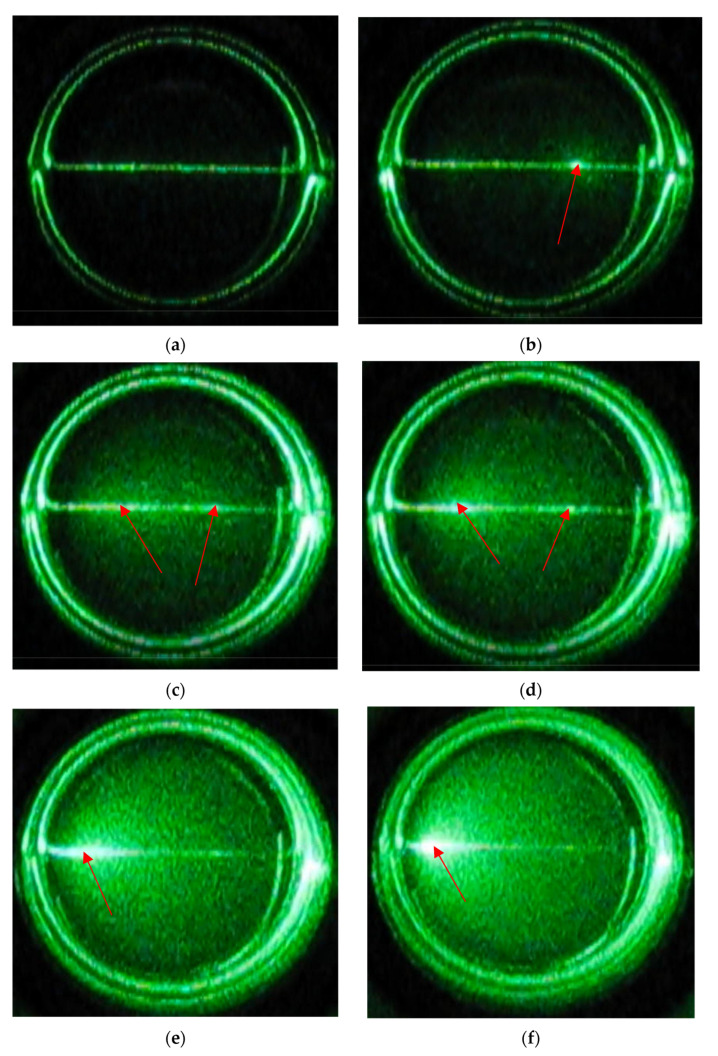
Sequence of images taken from a video recording of VIC-T polymerization and crosslinking of monomeric components under exposition to a laser light of 532 nm at 5 mW for 60 min; (**a**) is for 1 min of recording, (**b**) is for 6 min 50 s of recording, (**c**) is for 8 min of recording, (**d**) is for 8 min 35 s of recording, (**e**) is for 17 min 20 s of recording and (**f**) is for 55 min of recording. Arrows denote: green arrow in (**a**)—incident green laser light beam (valid for each photograph); polymerization is induced along the laser beam light; however, red arrows indicate some clearly distinguishable spots where the intensity of light was higher and thus polymerization was more advanced. High intensity of scattered light shifts towards incident beam over time of exposure.

The following experiment was designed to observe the white line formation process in the VIC-T in real time. To this end, a camera was attached above the VIC-T cuvette in the SLS instrument vat (Figure 1) to record a film during the exposure of the VIC-T to green laser light. The results in the form of a video are attached to this work as a supplementary material (Supplementary Material 1). Snapshots from the film were taken to illustrate the critical moments of polymerization and cross-linking and the LLT process in the VIC-T (Figure 4a–f). The laser beam passing through the VIC-T can be easily seen as a green glowing line in each picture. In Figure 4a, this line is quasi-uniform along the path of the laser beam. For the initial irradiation period up to about 3 min, the line looks like in Figure 4a. During this time, the SLS instrument shows a stable signal with about the same scattering amount of the green laser light. In a longer time, brighter spots appear along the laser beam (Figure 4b–d) and the SLS instrument shows a sharp exponential increase in the scattering amount (see Figure 10a in [16] for VIC-T; the figure is analogous to Figure 2a in this work for VIC-T without HQ). After this huge increase in the amount of scattered light registered by the SLS instrument, the scattering intensity dropped sharply to very low values, as can be seen in Figure 4e,f. In these photos it is easy to see that the intensity of the scattered light is currently highest near the entrance of the incident green laser light into the VIC-T cuvette. There was a large spot that shines very intensively. The light scattered by this large spot at the entrance of the laser beam illuminates almost the entire VIC-T in the cuvette. This will affect the VIC-T gel in areas where the beam has not passed through-discussed in the next section. The SLS instrument collects the quanta of scattered light at right angle relative to the center of the sample. Careful observation with the naked eye shows that the white line does not shine as brightly at this point at such a long time in comparison to the line visible in Figure 4a. A further conclusion is that the laser beam is trapped by scattering by large macromolecules growing along the beam path, and the longer the time is, this effect of growing macromolecules is transferred towards the entrance of the beam into the cuvette. This answers the question related to the nature of the LLT phenomenon.

**Figure 5:**
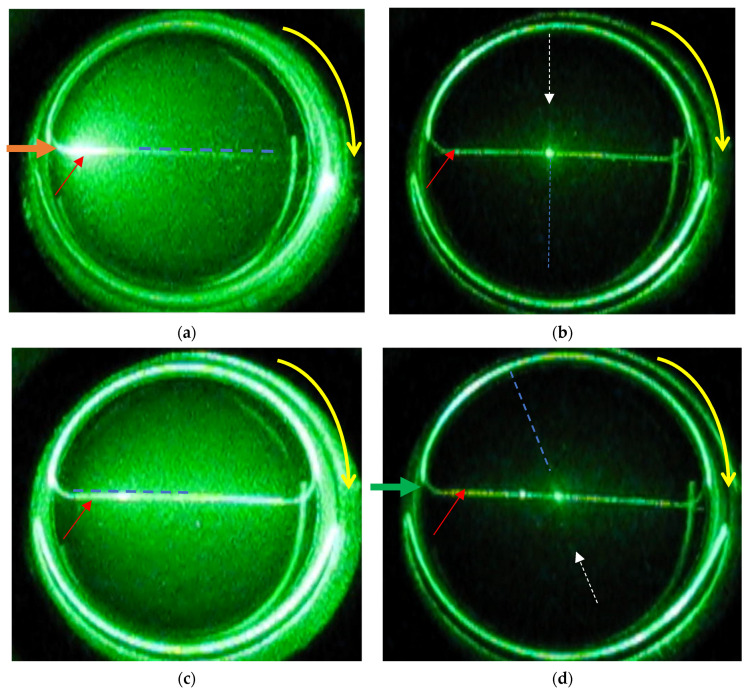
Sequence of images taken from a video recording of VIC-T polymerization and crosslinking of monomeric components under exposition to a laser light of 532 nm at 5 mW for 60 min. VIC-T sample after irradiation was left in a goniometer and it was rotated clockwise (indicated with yellow arrow): (**a**) is for the sample at 0° with respect to original position, (**b**) is for the sample at 90° with respect to original position, (**c**) is for the sample at 180° with respect to original position and (**d**) is for the sample at 250° with respect to original position. Arrows and line in the photographs denote, as follows: red arrow—laser beam passing through the sample; blue dash line—a prolongation of the white line corresponding to polymerized and crosslinked monomers; white dash arrow points to the white line as the blue line does; orange arrow in (**a**)—incident green laser light beam. Note that the white line corresponding to the polymerized monomers of VIC-T is hardly visible; therefore, it was marked with the blue dash line.

We were curious if the polymerization and cross-linking of the monomers in the VIC-T along the path of the laser beam is really inhomogeneous and if it is shifted towards the entrance of the laser beam into the cuvette after a longer exposure time. For this reason, the VIC-T cuvette was turned clockwise after approximately 60 min of exposure and a film was recorded (Supplementary Material 2). Then, several snapshots were taken, which are shown in Figure 5. In these snapshots, the dashed blue line and the white dashed arrow indicate the position of the white line corresponding to polymerized VIC-T monomers. Red and orange arrows indicate the path of the laser beam. By turning the cuvette, the position of the white line changed. In Figure 5a, the white line runs along the beam path exactly at the end of irradiation; strong illumination is visible at the entrance of the laser beam to the cuvette. In Figure 5b, this line is at the right angle to the incident laser beam. The illumination of the entire substance of the gel dosimeter is significantly reduced and only in the center of the sample is a bright spot visible as a result of the crossing of the laser beam with the white line. In Figure 5c the white line is at an angle of 180° to the incident laser beam. The entire gel dosimeter is illuminated by quanta of scattered light. Note that in this position the dosimeter illumination is more homogeneous than in the position in Figure 5a. This position shows that the degree of polymerization and cross-linking of the monomers is not the same further away from the entrance of the laser beam into the VIC-T cuvette. If it was the same, a bright spot should be visible near the entrance of the laser beam into the cuvette. In Figure 5d the white line is at an angle of 250° to the laser beam, so it interferes with the laser beam only in the center of the sample-a bright luminous point. In conclusion, only the white line, and thus the polymerized VIC-T monomers, contribute to the intense scattering of the incident laser light. The line is not homogeneous along its entire length.

### 3.2. Raman Spectroscopy Analysis

**Figure 6:**
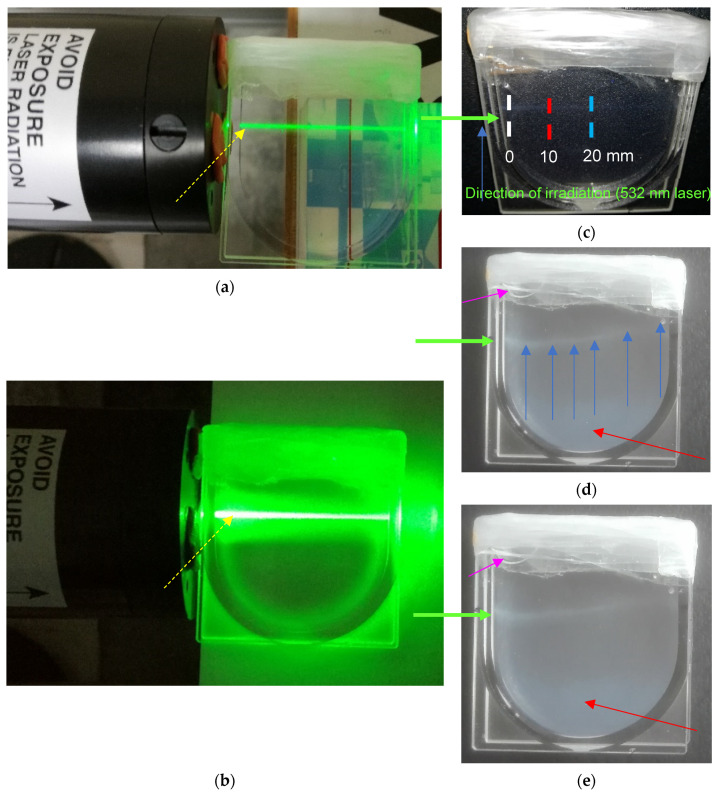
Visualization of the experiment reflecting irradiation of VIC-T in a flat quartz cuvette with green laser light of 532 nm (2 mW). The photograph in (**a**) corresponds to the irradiation at 0 min. In (**b**) is the same cuvette after 60 min irradiation. Yellow dash arrow is to point the region where polymerization and crosslinking took place during the irradiation. A thin line of the laser beam passing through the cuvette in (**a**) means no polymerization at the beginning of irradiation; however, a broader line of the laser beam in (**b**) denotes scattering of the laser light on large polymer molecules formed during irradiation. The photographs (**c**–**e**) illustrate the cuvette with VIC-T after irradiation: (**c**) immediately after 60 min irradiation, (**d**) 139 h after irradiation and (**e**) 215 h after irradiation. In (**c**) VIC-T in the cuvette is transparent and only a very thin white line can be spotted that corresponds to a polymerization and crosslinking effect; the line goes straight through VIC-T. In (**d**) this line bends upwards at a distance of around 10 mm (indicated by the blue arrows) and also the whole VIC-T volume converts to opaque (indicated in red arrow); in (**e**) self-polymerization is in progress, bending of the line is more profound. The opaqueness corresponds to a post effect occurred in VIC-T that was induced by the laser light highly scattered on the large molecules on the laser light line passing though the sample. In (**c**) the white, red and blue dash lines perpendicular to beam passing through the sample indicate the regions in which Raman spectroscopy was performed. Lilac arrow in (**d**) and (**e**) indicates a gel dosimeter concaved as a result of e.g., a bubble appeared over time after preparation or/and shrinking of polymer macromolecules formed in the whole dosimeter volume.

In order to track the photochemically induced reactions in VIC-T, an experiment was designed in which the VIC-T dosimeter was placed in a flat cuvette adapted to the measuring cavity of Raman spectroscopy instrument (Figure 6). The dosimeter in the cuvette was placed close to the green light output of the laser used (Figure 6a,b) so that the light beam passed through the dosimeter close to the upper wall of the cuvette. Approximately 4–5 min of initial illumination did not visually change the gel in the cuvette (Figure 6a). The beam was passing though the gel dosimeter such that the intensity of the green light along the laser beam was the same (visual inspection). After a long time, the green laser beam inside the gel dosimeter began to glow more intensely along its path. After 60 min of laser irradiation, the entire gel dosimeter and the beam in its path glowed with intense green light; the beam itself glowed even white–green in the center along the path through the cuvette (Figure 6b). It was assumed that polymerization and cross-linking of VIC-T monomers took place along the laser path, and the resulting large macromolecules scattered the laser light so that the entire dosimeter glowed green. All the same, the entire gel dosimeter in the cuvette was exposed to quanta of scattered green laser light. The naked eye examination also showed that the illumination along the laser path was more intense near the light entry into the cuvette (Figure 6b). This was analogous to the results presented in the previous section.

Immediately after irradiation, the gel dosimeter was crystal clear and transparent, and only a thin white line was visible in the part through which the laser beam was passed (Figure 6c). We will continue to refer to that white line in this section. The line ran exactly along the path of the laser beam. This white line corresponded to the polymerized monomer components of VIC-T. However, as the storage time passed, we noticed that the gel dosimeter changed from transparent to opaque, with the white line deviating from the initial linearity (Figure 6d,e). The opacity of the volume of the gel dosimeter seems to be a consequence of absorption of laser scattered quanta by monomers in the VIC-T, which must have triggered the polymerization process. It should be mentioned that our previous studies have shown that VIC-T is stable for at least 10 days after preparation with no visible change in transparency; measured R_2,0_ NMR (nuclear magnetic resonance, NMR, spin-spin relaxation rate; R_2_ = 1/T_2_, T_2_ denotes spin-spin relaxation time) was stable over the period mentioned (see Figure 3a,c in [16]).

The thin white line in the gel dosimeter bends downward at the beginning and upwards at a higher distance from where the laser beam has entered the cuvette (Figure 6d,e). There can be many reasons for this behavior. First of all, it should be emphasized that the cuvette was completely filled with the VIC-T dosimetric solution and tightly covered with a thick layer of Parafilm^®^ foil. As a result, fresh air hardly penetrates the gel during storage. The bending of the lines could be caused by polymer macromolecules forming in the entire volume of the dosimeter, which could shrink and thus distort the white line. Another reason may be the concavity of the gel on top due to the shrinkage of the polymer or/and the presence of a small bubble that has expanded during storage due to the contraction of the gel.

**Figure 7:**
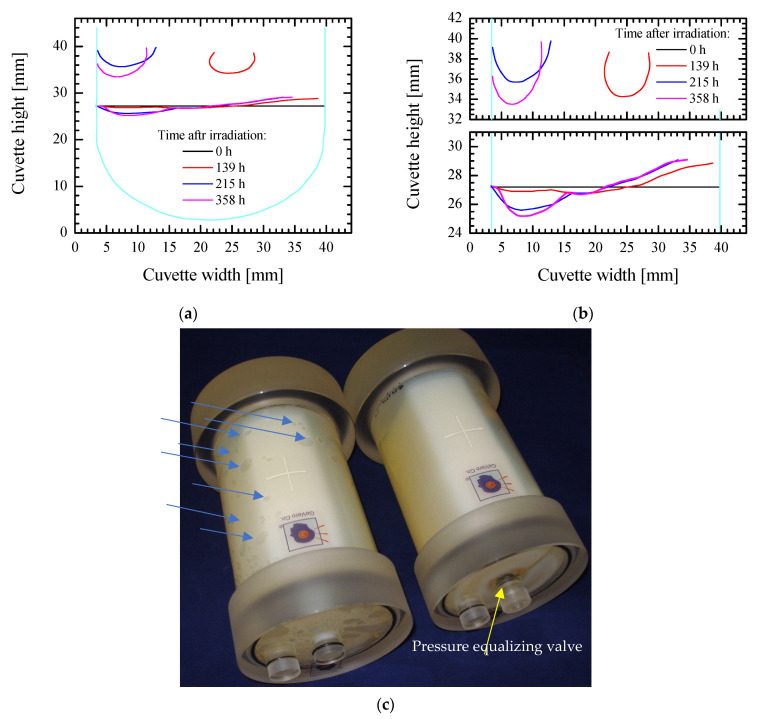
Evolution of the white line in the VIC-T dosimeter (see Figure 6c–e) corresponding to the polymerized monomers when exposed to green laser light (532 nm, 2 mW) (Figure 6a,b). The results obtained during storage relate to the autopolymerization of the entire volume of the VIC-T dosimeter after absorbing green laser light quanta scattered from the white line polymer (**a**,**b**). The three parabolic lines at the top of the graph correspond to the concave areas at the top of the VIC-T cuvette. Below are the lines corresponding to the white line of polymerized VIC-T monomers. At 0 h the white line was straight; in long times the line was bending. The cyan lines correspond to the walls of the cuvette; (**a**) the graph represents the dimensions of the entire cuvette; (**b**) the graph is drawn with a different OY range for better inspection of the white line bend. In (**c**), an exemplary polymer gel dosimeter (VIP) in two nearly identical containers (GeVero, Co., Poland) is shown after irradiation (ionizing radiation): the left container is without a pressure equalizing valve; the right container is equipped with an internal pressure equalizing valve. The blue arrows indicate the number of cavities formed during storage in the left valveless container that are not present in the valve container.

To look at changes in the gel dosimeter over time, several images of the VIC-T cuvette were taken after irradiation. The photographs were analyzed in ImageJ and the results obtained are shown in Figure 7. We could see that the bending of the white line corresponding to the polymerized VIC-T monomers at a distance of about 8 mm may be related to the concavity of the gel dosimeter at the top of the cuvette. This concavity at about 8 mm progressed with storage, while the other at about 25 mm disappeared with prolonged storage. The progress of the former is consistent with the bending of the white line at a short distance. The concavity of the gel dosimeter may result from the fact that the thick layer of Parafilm^®^ film is stiff, as is the cuvette used, which is a rigid container, and the contraction of the gel dosimeter forces such observed deformations (concavities). We saw a similar behavior of a 3D gel dosimeter (VIP) containing NVP in a large rigid container (left photo in Figure 7c) in another study. In the event that such a container is not equipped with an internal pressure equalizing valve, a number of empty cavities (indicated by blue arrows in Figure 7c) will be created. However, if the container is equipped with an internal pressure equalizing valve (indicated by the yellow arrow in the photo on the right), no cavities are present (Figure 7c).

The results obtained in this experiment led to the following conclusions related to the measurements of some 3D polymer gel dosimeters with optical computed tomography instruments equipped with green laser light: (i) scanning laser light can induce polymerization and crosslinking of monomer components for dosimetric systems containing gelatine and THPC; (ii) photochemically induced polymerization occurs not only along the path of the laser beam after a certain period of exposure of the dosimeter to laser light; large molecules formed along the path of a laser beam scatter laser light so intensely that the light quanta can be absorbed by the monomer molecules in the whole dosimeter, leading to polymerization in the dosimeter volume over an extended period of time; (iii) there is a maximal period for which a gel dosimeter with gelatine and THPC can be measured or scanned by optical techniques using a green laser without inducing polymerization of monomers; this period is related to the measurement temperature–at lower temperature it is longer, at higher temperature it is shorter and usually amounts to a few minutes (see Figure 10 in [16]); (iv) a construction of containers for 3D dosimeters impacts on a gel dosimeter substance; rigid containers promote the formation of empty cavities or concave places as a result of shrinkage of the gel dosimeter after irradiation, while containers with a pressure equalizing valve prevent such deformations of the gel dosimeters; they should promote the quality of the scanned images.

**Figure 8:**
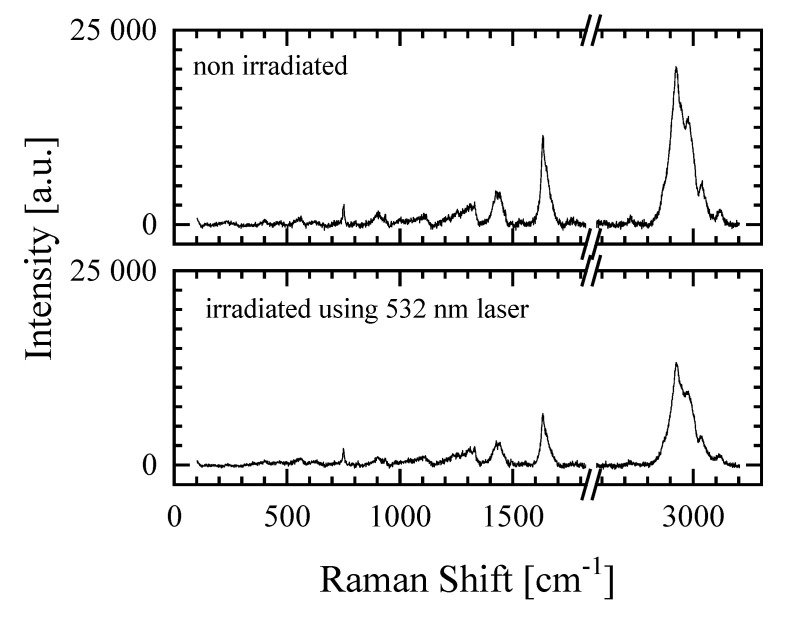
Raman spectra of VIC-T polymer gel dosimeter in non-irradiated and irradiated part. The irradiation was done by using 532 nm green laser light (2 mW) for a period of 60 min, which caused the formation of a white line corresponding to polymerized monomer molecules (see Figure 6c).

**Figure 9:**
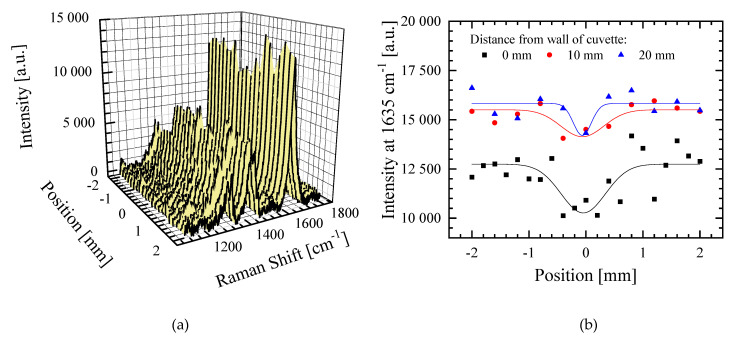
(**a**) Raman spectra recorded along the distance of 4 mm perpendicular to the white line corresponding to polymerized monomer molecules of VIC-T. The spectra start in a non-irradiated region of VIC-T, through green laser light irradiated region to non-irradiated region. (**b**) Intensity profiles of the bands at 1635 cm^−1^ versus the positions, where 0 mm denotes the middle of the white line (see Figure 6c) corresponding to the polymerized monomer molecules of VIC-T.

The area of the VIC-T dosimeter affected by green laser irradiation (532 nm, 2 mW) was analyzed by Raman spectroscopy. Figure 8 shows the Raman spectra for the non-irradiated and irradiated area of the dosimeter. As reported in our previous study [16], irradiation with ionizing radiation (30 Gy) reduces the intensity of the bands (in particular, the 1632 and 1647 cm^−1^ bands corresponding to C=C of *N*,*N*′-methylenebisacrylamide and *N*-vinylpyrrolidone, respectively) as a result of polymerization and cross-linking of monomeric VIC-T components. The same changes in the Raman spectra were observed by exposing the VIC-T to the green light of a 532 nm laser (Figure 8). A series of Raman spectra were registered from the non-irradiated part of the dosimeter through the irradiated (the white line) and towards the non-irradiated to a distance of 4 mm (Figure 9a). This three-dimensional graph illustrates the intensity variation of some bands for the area of laser irradiation for a distance of approximately ±1 mm. In Figure 6c dashed lines represent three positions perpendicular to the white line corresponding to the polymerized monomers induced by green laser light (60 min irradiation) for which profiles derived from Raman spectra were prepared (Figure 9b). The profiles correspond to the vinyl group C=C and R-NH-CO group of MBA (1635 cm^−1^).

The profiles clearly show the area of conversion of monomers into polymer under the influence of irradiation with green laser light–the white line. The profiles obtained were deconvoluted using the Gauss function (Figure 9b). Full width at half maximum (FWHM) was calculated for the profiles to be 0.92, 0.84 and 0.39 mm at the following distances: 0 (near the incident laser beam input), at 10 and 20 mm, respectively. This means that the polymerized area along the white line is the widest near the entrance of the laser beam into the cuvette and narrows at greater distances. This corresponds well to the images showing the high intensity glowing of the VIC-T at shorter distances, which falls at greater distances from the beam entry into the cuvette (Figure 4e,f, Figure 5a and Figure 6b). The reason for this observation is the accumulation of the dose from the laser beam absorbed by the VIC-T at the beam entry into the cuvette due to the continued formation of large macromolecules in the path of the laser light. Growing macromolecules scatter the laser light that is trapped at the beginning of its path in the VIC-T (LLT phenomenon). This boosts the dose from the incident light and scattered light at the beginning of the laser light path, increasing the conversion of VIC-T monomers (widening of the profiles in Figure 9b) and formation of high molecular weight macromolecules that scatter even more laser light. Note that for distances greater than ±~1.1 mm (0 mm profile), ±~0.9 mm (10 mm profile) and ±~0.5 mm (20 mm profile), the intensity of 1635 cm^−1^ band increases, which corresponds to a higher concentration of C=C MBA bonds (all the same NVP C=C bonds) and thus a lower conversion to polymer of the vinyl groups outside the green laser light path. In summary, Raman spectroscopy measurements confirmed the LLT phenomenon occurring in the VIC-T dosimeter irradiated with green laser light. Basically, this involves the conversion of VIC-T monomers into high molecular weight polymer macromolecules that scatter the laser light, prevent the laser beam from penetrating the dosimeter, and accumulate the dose of laser radiation near the beam entering the VIC-T dosimeter. It should be noted that Raman analysis showed that the polymerization reaction induced by green laser light was similar to that induced by ionizing radiation [16]. The exact mechanism of polymerization and cross-linking of vinyl monomers specific to 3D gel dosimeters has been studied previously [10,25,26].

## 4. Conclusions

The aim of this work was to investigate and explain the phenomenon observed in the VIC-T gel dosimeter irradiated with green laser light. The phenomenon was that the laser light was trapped by the gel substance of the dosimeter. Therefore, it is called the laser light trapping (LLT) phenomenon. Two questions were posed: (i) what VIC-T components contribute to the LLT and (ii) what is really behind the LLT. The main conclusions of this work are as follows: (i) gelatine and THPC components of VIC-T are required for the occurrence of the LLT; (ii) irradiation of the VIC-T with green laser light causes polymerization and cross-linking of the monomeric components of the VIC-T; (iii) growing macromolecules along the path of the laser light cause the laser light to scatter and the laser dose to accumulate closer to the entry of the laser beam into the VIC-T cuvette; (iv) the highly polymerized region at the entrance of the laser beam into the cuvette traps the laser beam that hardly passes it, and the scattered light delivers radiation quanta to the whole gel dosimeter substance; (v) a consequence of the latter is that polymerization is observed throughout the dosimeter after prolonged storage; (vi) scanning of 3D dosimeters with gelatine and THPC in the composition is likely by means of optical computed tomography with green laser light, however the scanning time (exposure to green laser light) of the dosimeter should be less than 3 min and finally (vii) shrinkage of the VIC-T was observed, which is a natural process that may occur in other 3D polymer gel dosimeters, likely resulting from shrinkage of macromolecules formed; this leads to some voids that can distort the scan images (from any 3D scanning technique); this can be avoided if a gel dosimeter container is equipped with a pressure equalization valve.

Based on other unpublished observations, it is suspected that the LLT phenomenon may occur in other polymer gel dosimeters irradiated with a shorter wavelength of the laser. Therefore, the LLT phenomenon requires further research, both with irradiation with lasers of different wavelengths of light, and with the use of different dosimetric compositions to broaden the conclusions drawn in this paper.

## Data Availability

The data supporting reported results is not stored in any publicly archived datasets. The readers can contact the corresponding author for any further clarification of the results obtained.

## Supplementary Materials

The following are available online at https://www.mdpi.com/article/10.3390/ma14143961/s1, Supplementary Material 1 and Supplementary Material 2.
